# The effect of reverse intergenerational support on the happiness of Chinese older adults: A moderating effect analysis based on role conflict

**DOI:** 10.3389/fpsyg.2022.1080772

**Published:** 2023-01-10

**Authors:** Hongfeng Zhang, Shunyi Li, Wenwen Dai, Hanning Wang

**Affiliations:** ^1^School of Economics, Guizhou University of Finance and Economics, Guiyang, Guizhou, China; ^2^School of Economics and Trade Management, Changzhou Institute of Light Industry Technology, Changzhou, China; ^3^School of Big Data Application and Economics, Guizhou University of Finance and Economics, Guiyang, Guizhou, China

**Keywords:** intergenerational exchange, intergenerational support, reverse intergenerational support, role conflict, happiness, China

## Abstract

Intergenerational support is bidirectional, and reverse intergenerational support refers to parents providing financial support, time support, and spiritual support to their offspring. The emergence of reverse intergenerational support has created role conflicts among different groups of older adults. Based on survey data from 3,170 elderly people in eight sample provinces in China, this paper empirically investigates the relationship between reverse intergenerational support and the happiness of the elderly in contemporary China and the moderating effect of role conflict in it, using an ordered logit model. It was found that, first, reverse economic support reduces the happiness of the elderly, and reverse time support and reverse spiritual support can significantly enhance the happiness of the elderly. Second, in the presence of role conflict, the effect of reverse time support and reverse spiritual support on the enhancement of older adults’ happiness was suppressed; in the presence of role enhancement, the effect of reverse economic support on the reduction of older adults’ happiness was mitigated. The above findings provide new empirical evidence for understanding the relationship between reverse intergenerational support and the happiness of the elderly, which is prevalent in contemporary China, and offer new insights for enhancing happiness.

## 1. Introduction

Happiness, as a subjective feeling, reflects people’s quality of life and the national welfare they enjoy, and has become the focus of social and economic policies (Mackerron, 2011; Andrada-Alexandra, 2016). Population aging is an important trend in China’s social development and the basic national conditions of China for a considerable period in the future. In this context, national strategic goals and measures such as “implementing the national strategy to actively cope with population aging” and “reaching a new level of people’s livelihood” have been put forward successively. At the same time, China’s intergenerational relations are changing. The traditional phenomenon of “filial piety” is gradually replaced by the “reverse intergenerational support” phenomenon of “parents helping their children.” Under the transformation of intergenerational relations in contemporary China and the major national concern about the happiness of the elderly, it is of great practical significance to study the relationship between reverse intergenerational support and the happiness of the elderly.

The intergenerational model in contemporary China has changed from the traditional feedback model (Fei, 1983), to a reciprocity model (Xiong, 1998), and an exchange mode (Wang, 2008). In the exchange mode, the intergenerational relationship has a bidirectional feature. Drawing on Caldwell’s (1976) definition of intergenerational transfer, reverse intergenerational support refers to the economic support, time support, and spiritual support of the offspring. On the one hand, the reverse intergenerational support in contemporary Chinese society is not only widespread (Wu, 2015), but also unique and different from western society (Hansen and Svarbered, 2011);on the other hand, when “social participation” advocated in active aging and the “intergenerational participation” in reverse intergenerational support are superimposed, the social roles of the elderly will be more diversified, and the elderly will face more role conflicts, and the opposition and conflict between the roles will lead to psychological conflicts within the elderly (Xi, 2010). So, how does reverse intergenerational support, which is prevalent in contemporary China and has a unique cultural background, affect the happiness of the elderly? Does the relationship between reverse intergenerational support and happiness change in the elderly group with role conflict? The answers to these questions are important factors that cannot be ignored to improve the happiness of the elderly in China.

Compared to the existing studies, this paper contributes as follows: First, the research perspective is changed from the positive intergenerational support from the offspring to the parent in the traditional feedback mode to the reverse intergenerational support to the offspring in the contemporary exchange mode, and it analyzes the relationship between the reverse intergenerational support and the happiness of the elderly, enriching the factors influencing the happiness of the elderly; Second, the introduction of role conflict into the analysis of the elderly’ happiness is an innovative exploration that focuses on the role conflict older adults may face in the process of reverse intergenerational support provision and its role in the happiness of the elderly, which is conducive to noticing the realistic needs of the elderly with different characteristics to promote a more balanced and full realization of the good life of contemporary older adults.

## 2. Literature review

The study of reverse intergenerational support follows the process of research, from exploratory research to descriptive research to explanatory research. From the early discovery of the existence of reverse intergenerational support (Grundy, 2005; Albertini et al., 2007; Song and Qi, 2011), to the description of its characteristics by the later period (Fingerman et al., 2015; Huang and Duan, 2018; Huang et al., 2018), then to the current reverse intergenerational support for the three generations in various aspects of the impact and mechanism interpretation (Li, W. X. et al., 2021; Zhang et al., 2021), as a new phenomenon in the process of social transformation, reverse intergenerational support has attracted more and more people’s attention. Current research generally divides intergenerational support into three aspects: economic support, time support, and spiritual support (Bai and Gu, 2021; Hou et al., 2021); accordingly, this paper divides reverse intergenerational support into three aspects: reverse economic support, reverse time support, and reverse spiritual support. Focusing on reverse intergenerational support and the happiness of the Chinese elderly, existing studies mainly have the following three opinions:

First, reverse intergenerational support improves the happiness of the elderly. In the aspect of reverse economic support, some studies have found that the economic support provided by the elderly to their children can enhance the sense of self-efficacy and fairness of the elderly and then improve life satisfaction (Zhang and Li, 2005; Zhen and Merril, 2008; He, 2011). In terms of reverse time support, some studies have shown that there is a strong positive relationship between the intergenerational care of grandchildren and the life happiness of the elderly, which means that intergenerational care is an important link to enhance the intergenerational connection between paternity and offspring. An important manifestation of active aging is to make it easier for older people to discover the meaning of life and realize their self-worth (Powdthavee, 2011; Zhang and Chen, 2014; Arpino et al., 2018; Cheng and Jiang, 2019). In terms of reverse spiritual support, the elderly provide rich and powerful emotional support to their children to show their wisdom and reflect on their value, to enhance their sense of self-affirmation and satisfaction (Kuause and Shaw, 2000).

Second, reverse intergenerational support reduces the happiness of the elderly. In terms of reverse economic support, the parents provide reverse economic support for the offspring for a long time, which will inevitably increase the economic burden of the parents and thus reduce the happiness of the elderly (Wu, 2015). In terms of reverse time support, the elderly are prone to boredom and frustration if they provide more life care services, which increases their health risk and reduces happiness (Chambers et al., 2001; Ates, 2017). In terms of reverse spiritual support, if the parents still need to give too much attention and help to the adult children, it will be regarded as a failure of education and then increase the psychological pressure on the parents (Schwarz et al., 2010).

Third, the role of reverse intergenerational support on elderly happiness depends on a more detailed classification. The difference in household registration, liquidity, and income will cause a difference in the role of happiness in the elderly (Jin and Liu, 2017; Zheng et al., 2018). The age difference has also made the reverse time to support the effect of the old man’s happiness in different age groups (Wang and Chen, 2019; He et al., 2020). In terms of living arrangements, when it is an elderly couple or a single elderly person who lives with their children, the happiness of the elderly couple increases, and the happiness of the single elderly person decreases. The reason for these differences is the shift in the “center of power” of the family or not (Huang and Duan, 2018). When providing reverse intergenerational support, whether the elderly have received higher education (Mahne and Huxhold, 2015), whether they have become grandparents for the first time (Di Gessa et al., 2020), whether they have a close family relationship (Sun and Dutta, 2021), and whether they are still participating in work (Arpino and Bellani, 2022), which will become the influencing factors of whether the elderly can improve their happiness when providing reverse intergenerational support.

It can be seen from the existing research results of reverse intergenerational support and the happiness of the elderly that First, there are differences or even opposite conclusions in the existing empirical findings, which may be caused by the differences in age, region, and research subjects. In particular, China is experiencing a sharp development and change from a traditional agricultural society to a modern industrial society. This transformation has had a significant impact on various aspects of contemporary Chinese society, including the modern market economy and changes in family structure. Therefore, China must make retest the “contemporary” and “Chinese” elderly people in reverse intergenerational support and their happiness as China enters a new stage of development. Second, with the continuous change in Chinese society, more heterogeneous analysis was introduced in the later investigation of reverse intergenerational support and the happiness of the elderly. The reason is that elderly people with different characteristics differ in their economic ability, time conditions, health status, and internal and external expectations when providing reverse intergenerational support. Jin and Liu (2017) and Zheng et al. (2018) found that with the same reverse time support for providing grandson generation care, the rural elderly are more faced with the integration problem with their children’s families, communities, and cities, so their happiness is no longer significantly improved.

The existing literature forms a good research base and gives useful insights: on the one hand, there is a need to focus the research object in a specific cultural and social context, on the other hand, the analysis of heterogeneity around older adults is rich enough, and it is necessary to refine and summarize the factors that cause differences. Role conflict theory provides a good perspective to explain the differences in the happiness of different older age groups in reverse intergenerational support. Role conflict theory suggests that conflict arises when individuals have different roles and personal expectations in different arenas (Merton, 1967) and that stress arises or increases when each individual, including the elderly, has a series of conflicting role obligations (Goode, 1960), and happiness is compromised (Laura et al., 2020). Arpino and Bellani (2022) found that the provision of grandchild care tends to be beneficial for grandmothers’ well-being only if they do not combine this activity with paid work. What forms a complementary relationship with the role conflict theory is the role enhancement theory, which holds that the integration of multiple roles can enhance happiness because the elderly can obtain a sense of self-satisfaction from a variety of integrated roles (Moen et al., 1995). Role conflict theory has been widely applied to the examination of different identities, such as teachers, civil servants, women from poor families, journalists, etc., and extended to larger areas such as national politics, social work, corporate governance, etc. Li L. Y. et al. (2021) made a theoretical analysis of the role conflicts faced by Chinese elderly people, but in general, there are relatively few studies on the role conflicts faced by contemporary Chinese elderly people.

Given this, this paper uses an ordered logistic model through primary data collection to examine the role of three different types of reverse intergenerational support on the happiness of contemporary Chinese older adults, and further divides older adult groups under the perspectives of role conflict and role enhancement to examine the influence mechanisms of role conflict and role enhancement in it.

## 3. Theoretical development and hypothesis

Under the background of the rapid changes in Chinese society, intergenerational relations and their theories are also constantly evolving. On the one hand, China’s family change dynamics present modernized features consistent with Western family modernization, such as core families, miniaturization of the first marriage, etc. On the other hand, it also shows the family model in Chinese traditional culture, such as the long-term existence of filial piety culture, strong intergenerational relations, and the relatively high proportion of multi-generation families (Ji, 2019). Fei (1983) believes that the relay relationship between China and the West is different. The west is generation after generation, which means the parents raise the children, and the children raise the grandchildren. The intergenerational relationship in China is more in line with the characteristics of the feedback relationship, that is, the parents nurture the offspring, and the offspring support the parents. Since ancient times, it has been achieved because the parents have achieved commonality in the control of resources and the interests of each generation (Liu, 2016). With the advancement of the reform process and the continued progress of social transformation, the control of the family’s resources is transferred from the parent to the child, and the realization of the feedback mode is destroyed and evolved into exchange modes (Miao, 2020). Although in the exchange mode, the intergenerational relations of Chinese society have rational characteristics (Cai, 2015), intergenerational exchange relationships are not equivalent exchanges, but a process of mutual assistance and reciprocity (Wang, 2008).

With the continuous evolution of intergenerational relationships, the role of the elderly is also evolving, and the conflict of older characters is coming. The role conflict theory provides a theoretical logic and a research perspective for analyzing the happiness of contemporary Chinese elderly people. Based on the traditional Chinese ethics of filial piety and considering the characteristics of an active aging society, the role specification and role expectation after the parent enter the elderly role is that the parent hopes to get the support of children in a good intergenerational relationship, and the old are happy (Bai and Wang, 2021). The role positioning mainly includes three aspects: the parent expects to support their children; the parent hopes to have good intergenerational relations, and the parent hopes to achieve active aging.

First, the parents expected the support of their children. Although the concept of raising children in old age has been diluted, the role of children is still an expectation of the elderly, and family pension is still a common pursuit and dependent way (Bai, 2021). If there is a continuous situation of being “living off their parents,” on the one hand, continuous reverse economic support for children will cause a conflict with economic support ability; on the other hand, it will conflict with the role of the parents who want to be supported, so reverse economic support will cause a decline in the happiness of the elderly.

Second, parents want a good intergenerational relationship. In modern times when the family scale is shrinking, taking care of grandchildren is not only the biggest spontaneous support of many elderly people for their children but also the expectation of the family and society for older parents (Li L. Y. et al., 2021). Intergenerational relations, intergenerational interaction, and intergenerational communication are the main sources of spiritual support for elderly parents. The connotation of intergenerational relations is the communication and interaction between two generations (Feng, 2020). Therefore, reverse time support and reverse spiritual support can improve the happiness of the elderly. Based on the above analysis, the specific hypothesis was as follows:

Hypothesis *1a*: Reverse economic support reduces happiness among older people.

Hypothesis *1b*: Reverse time support improves the happiness of the elderly.

Hypothesis *1c*: Reverse spiritual support improves the happiness of the elderly.

Third, parents want to age actively. Under the background of active aging, health, participation, and security are the three core elements. Health elements include not only physical health but also mental health; security includes not only social security but also family security. All of these are the roles and expectations of the elderly in contemporary China in the context of active aging. Because of the social context of transformation and the evolving intergenerational relationship, the elderly are inevitably confronted with a role conflict between reality and expectation. Referring to the existing classification criteria of role conflict by Li L. Y. et al. (2021), older people face expectation conflict, ability conflict, time conflict, and behavior conflict.

Expectation conflict. First, there is an expectation conflict regarding the elderly’s role positioning. Contemporary society as a whole still highly expects older parents to give priority to taking care of their children’s families and providing care for their grandchildren, which conflicts with the expectation that the elderly will have leisure time to participate in social and recreational activities after retirement. Second, regarding the conflict of mutual expectations in two-way intergenerational support, with the traditional responsibility and obligation of supporting parents on one side and the normative phenomenon of economic and non-economic “gnawing” on the other side (Liu et al., 2022), it is difficult to avoid the conflict between the intergenerational support expectations of the father’s and the children’s generations, given the limited intra-family resource allocation after the heavy cost of living distortion (Li et al., 2019). Third, there is a conflict of expectations regarding the allocation of reverse support for multiple children. It is a common social phenomenon that elderly parents are always considering which child to provide more reverse economic support to, and struggling with which child to provide grandchild care to, and even in the case of elderly couples going to one child each to take care of their grandchildren to show fairness. This leaves older couples separated for a longer period, and the paternal generation often chooses to give up their expectations in the conflict to meet the expectations of their children (Li et al., 2019).

Ability conflict. First, the conflict of reverse economic support capabilities. If the parents are required to provide continuous reverse economic support to the adult offspring with limited financial capacity, it will weaken their ability to provide old-age security and reduce their sense of security in old-age security (Chen et al., 2022). Under China’s urban–rural dichotomy, reverse economic support is more likely to form a conflict of ability for rural elderly who are relatively economically weaker. Conversely for Chinese older adults with more resources, the more power their families have, the more reverse economic support they have (Xin and Shuying, 2020); secondly, there is a conflict regarding the ability of reverse time support. Intergenerational parenting is intense, and caring for infants and toddlers aged 0–3 years is even more so. This creates a capacity conflict between older and weaker fathers, which is the main reason why many fathers have “retreated” from their children’s second-child births in recent years after participating in the care of their first grandchild (Zhong and Guo, 2017).

Time conflict. First, there is a time conflict between the life care of children and the social participation and social activities of the elderly. Although the health status of people aged 60–70 is better than that of people over 70, from the perspective of intergenerational age structure, it is precisely the people aged 60 and 70 who need to face the daily care of their younger grandchildren. This intergenerational support without respite creates a time conflict with social participation; second, the conflict between life care for grandchildren and willingness to postpone retirement. The transformation of society has caused the willingness of adult children to be planned according to their parents’ retirement time (Feng et al., 2020). So, parents face a choice between having grandchildren or having a delayed retirement.

Behavioral conflict. Behavioral conflicts are more often seen in the old drifters who have moved to the city with them, and the conflicts formed in the process of caring for their grandchildren in terms of separation from their partners, estrangement from their offspring spouses, and integration with the urban community. In the temporary main family formed with their children in the city, they are redundant in the nuclear family, marginalized in the intergenerational relationship, foreigners in the regional conflict in daily life, and strangers in public life in community interaction (Xu and Hua, 2018; Li, 2022).

In addition to role conflict, there are role enhancements in reverse intergenerational support. Drawing on the type and division of role conflict, role enhancement can be reflected in four aspects: consistent expectation, consistent ability, consistent time, and consistent behavior. Research points of Feng and Fan (2020) out that elderly people with good economic conditions, good health conditions, and the only child have better pension ability and higher inner security. Under the urban–rural dual structure in China, the urban elderly people have better economic conditions than rural elderly people because they enjoy more sufficient pension guarantee and they also know more clearly about their children’s survival pressure. There are no role conflicts, and even a role enhancement is formed to a certain extent. Similarly, the reverse economic support of elderly people in good health and those with only one child appears to be more consistent in ability and expectations. Zhang et al. (2021) point out that when the parents receive support from their children, it significantly positively affects the reverse economic support for the parents to provide mortgage payments for their children, which also proves the exchange of the intergenerational relationship. In an exchange intergenerational relationship if the elderly receive positive intergenerational support from their children, then it is easier for the elderly to achieve the expectation consensus by providing the reverse economic support to their children.

In summary, the four types of role conflicts that older adults of different characteristic groups in China will face in the process of reverse time support and reverse spiritual support, as well as the role enhancement they face in reverse economic support, are summarized in Table 1. Guided by role conflict theory, Hypothesis 2 and Hypothesis 3 were obtained.

**Table 1:** Role conflict/enhancement in the provision of reverse intergenerational support by different groups of older adults.

Index	Group segmentation	Reverse economic support	Reverse time support	Reverse spiritual support
Household register	Countryside		Behavior conflict	
	Town	Role enhancement		
Age	60–69 years old		Expectation conflict	
Number of offspring	Only child	Role enhancement		
	Non-only child		Expectation conflict	Expectation conflict
Health condition	No chronic disease	Role enhancement		
	Chronic disease		Ability conflict	
Delay retirement	Continue working		Time conflict	
Positive intergenerational support	With positive intergenerational support	Role enhancement		
	No positive intergenerational support		Expectation conflict	Expectation conflict

Hypothesis *2*: Compared with the whole elderly group, the suppressing effect of reverse economic support on the happiness of the elderly subdivided group with role enhancement will be resolved.

Hypothesis *3*: Compared with the whole elderly group, the promotion effect of reverse time support and reverse spiritual support on the happiness of the elderly subdivided group with role conflict will be suppressed.

## 4. Methodology

### 4.1. Research design

In this paper, the happiness of the elderly is measured in 5 levels, i.e., “very unhappy = 1, not very happy = 2, average = 3, relatively happy = 4, very happy = 5.” Since the explained variables happiness is a multivariate discrete variable, an Ordered Logit Model is used for estimation. The model-specific form is as follows:


(1) $$Happiness_{i}=\alpha +\beta Reversesupport_{i}+\gamma Z_{i}+\pi_{p}+\varepsilon_{i}$$

$Happiness_{i}$ is the happiness of the elderly and is an observable scale variable expressed as an integer value from 1 to 5; $Reversesupport_{i}$ is the variable that provides reverse intergenerational support, including reverse economic support, reverse time support, and reverse spiritual support; $Z_{i}$ is the control variable, including the individual and family characteristics of the sample; $\pi_{p}$ represents the province virtual variable; is observation value; is the parameter variable value to be estimated; β is random disturbance items.

To verify hypothesis 1, this paper focuses on the magnitude, direction, and significance of *β*. In the part of robustness analysis, this paper will replace the explained variables, replace the sample data, and use a variety of econometric models to test the benchmark regression results. To verify Hypotheses 2 and 3, this paper will analyze the heterogeneity of the typical elderly population with role conflict and role enhancement, focusing on whether the action direction and significance of *β* value under heterogeneity has changed. It should be noted that since role conflict is a state whose direct measurement is difficult, this paper defines the scope of role conflict as a typical group that may have role conflict by drawing on the findings of existing relevant studies and by observing the changes in the relationship between reverse intergenerational support and happiness of these typical groups, to explain the inhibition of role conflict on happiness and the enhancement of role enhancement on happiness.

### 4.2. Data source and variable definition

#### 4.2.1. Data sources

Relying on the fund project, the project team collected 3,527 questionnaires from eight provinces from July 2021 to August 2022. This survey adopted the method of quota sampling, selecting eight provinces, including Jiangsu, Zhejiang, Guangdong, Hubei, Guizhou, Shanxi, Hebei, and Gansu, basically covering the east and the west and the south and the north, and fully taking into account the possible differences in sample characteristics in different regions. Excluding 87 samples aged 59 and below, 186 samples with missing values, and 84 samples without children, the final valid questionnaire was 3,170.

#### 4.2.2. Variable selection

Explained variable: In measuring the happiness of the elderly, the five-level scale is used to quantify their well-being, and the values of “very unhappy,” “not very happy,” “generally,” “relatively happy” and “very happy” are assigned to 1, 2, 3, 4, and 5, respectively. Explanatory variable: According to the definition of reverse intergenerational support in this paper, the explanatory variables are “reverse economic support,” “reverse time support,” and “reverse spiritual support.” Reverse economic support: the author uses “Do you need financial help from your children?” to measure; Reverse time support: the author uses “Do you need to help your children with housework or take care of your grandchildren?” to measure; Reverse spiritual support: the author uses “Will you take the initiative to care about your children’s lives and work, and give them encouragement or guidance?” to measure; Moderating variables: refer to Table 1 for moderating variables. This paper uses the factors that may have an impact on the role norms of the elderly as moderating variables, including household registration, age, number of children, chronic diseases, delayed retirement status, positive intergenerational support, etc. Control variables: based on previous studies, a series of internal and external characteristics variables that have been shown to influence the happiness of the elderly are added as control variables, including economic conditions, pension method realized (whether the ideal and realistic pension method are consistent), marital status, health status, community retirement service content, and gender (Xing and Hu, 2022). This paper further controls for regional fixation effects.

Since happiness is a subjective feeling, explanatory variables such as “happier than before” and “happier than the surrounding people” are also introduced in the corresponding models in the robustness test. Assign the values of “unhappy,” “uncertain,” and “happy” to 1, 2, and 3, respectively. The definition and descriptive statistics of each variable are shown in Table 2.

**Table 2:** The definition of variate and descriptive statistics.

Variate	Code and assignment			Min	Max	Mean	SD
**Explained variable**							
Happiness	1, Very unhappy 4, Relatively happy	2, Not very happy 5, Very happy	3, General	1	5	4.17	0.88
Compared with the previous sense of happiness	1, Not happy	2, Uncertain	3, Happy	1	3	2.49	0.66
Compared with the happiness of the people around you	1, Not happy	2, Uncertain	3, Happy	1	3	2.68	0.58
**Explanatory variable**							
Reverse economic support	0, No	1, Yes		0	1	0.22	0.41
Reverse time support	0, No	1, Yes		0	1	0.65	0.48
Reverse spiritual support	1, No	2, Uncertain	3, Yes	1	3	2.67	0.60
**Controlled variable**							
Economic condition	1, Not very good	2, Average	3, Very good	1	3	2.33	0.67
Pension method realized^*^	0, No	1, Yes		0	1	0.95	0.21
Marital status	0, No spouse	1, With spouse		0	1	0.73	0.44
Health condition	1, Not very good	2, Average	3, Very good	1	3	2.32	0.65
Community service	0–9, Number of items of community service content obtained			0	9	1.84	2.02
Gender	0, Male	1, Female		0	1	0.51	0.50
**Heterogeneity test variable**							
Household register	0, Agricultural	1, Non-agricultural		0	1	0.19	0.40
Age	1, 60-69 years old	2, 70-79 years old	3, 80 years old and over	1	3	1.62	0.72
Number of offspring	1, no kid	2, 1 kid	3, 2 kids 4, 3 or more children	1	4	3.14	0.84
Chronic disease	0, No	1, Yes		0	1	0.34	0.48
Delay retirement	1, no work	2, Occasionally work	3, Work	1	3	1.77	0.80
Positive economic support	1, No	2, Occasionally	3, Often	1	3	2.56	0.56
Positive time support	0, No	1, Yes		0	1	0.93	0.26
Positive spiritual support	0, No	1, Yes		0	1	0.68	0.47

* “Pension method realized” refers to whether the current pension method of the elderly, including home care, community care, and institutional care, is consistent with their expectations.

## 5. Empirical analysis

### 5.1. Benchmark regression

Column (1) of Table 3 reports the empirical results of reverse economic support, reverse time support, and reverse spiritual support on the happiness of the elderly. Specifically, the effect of reverse economic support on the happiness of the elderly was significantly negative at a 5% confidence level, indicating that reverse economic support decreased the happiness of the elderly. The effect of reverse time support on the happiness of the elderly was significantly positive at a 10% confidence level, providing weaker evidence to support that reverse time support is beneficial in enhancing the happiness of the elderly. The effect of reverse spiritual support on the happiness of the elderly was significantly positive at the 1% confidence level, suggesting that an increase in reverse spiritual support is beneficial in enhancing the happiness of the elderly. The above research conclusions verify Hypotheses 1a, 1b, and 1c, namely that reverse economic support reduces the happiness of the elderly, reverse time support improves happiness, and reverse spiritual support will improve the happiness of the elderly. According to the empirical results of each control variable, the economic conditions, whether the pension model is realized, health status, and community services can significantly improve the happiness of the elderly. However, the impact of marital status and gender on the happiness of the elderly did not have a significant protective effect.

**Table 3:** Results of base regression and robustness tests by replacing explanatory variables and measures.

	(1) Logit	(2) Happier than before	(3) Happier than others	(4) OLS	(5) Probit
Reverse economic support	−0.247^**^	−0.216^*^	−0.482^***^	−0.110^***^	−0.135^**^
	(−2.387)	(−1.938)	(−3.963)	(−2.984)	(−2.294)
Reverse time support	0.161^*^	0.264^***^	0.116^*^	0.054^*^	0.116^*^
	(1.793)	(2.775)	(1.916)	(1.648)	(1.916)
Reverse spiritual support	0.333^***^	0.322^***^	0.380^***^	0.148^***^	0.204^***^
	(4.853)	(4.386)	(4.779)	(5.919)	(5.258)
Economic condition	1.344^***^	1.342^***^	1.335^***^	0.494^***^	0.746^***^
	(18.514)	(18.003)	(17.886)	(20.075)	(18.715)
Pension method realized	0.814^***^	0.855^***^	0.846^***^	0.418^***^	0.516^***^
	(4.064)	(3.964)	(3.894)	(6.073)	(4.863)
Marital status	0.005	−0.022	0.015	0.027	0.020
	(0.052)	(−0.227)	(0.149)	(0.751)	(0.352)
Health condition	0.833^***^	0.872^***^	0.859^***^	0.296^***^	0.466^***^
	(11.743)	(12.000)	(11.793)	(11.749)	(11.638)
Community service	0.116^***^	0.118^***^	0.115^***^	0.032^***^	0.061^***^
	(4.930)	(4.909)	(4.777)	(4.154)	(4.761)
Gender	−0.100	−0.076	−0.091	−0.023	−0.047
	(−1.161)	(−0.885)	(−1.061)	(−0.749)	(−0.949)
Regional virtual variables	Control	Control	Control	Control	Control
*N* (number of observations)	3,170	3,170	3,170	3,170	3,170
*R*^2^	0.1637	0.1752	0.1595	0.3121	0.1606

^***^p < 0.01; ^**^p < 0.05; ^*^p < 0.1, as in the following tables.

### 5.2. Robustness analysis

To verify the robustness of the model, this paper uses a variety of methods. First, replace the explained variable. Considering the sense of happiness generally refers to people’s subjective evaluation of the current satisfaction of life (Diener, 2000), and when comparing the actual living conditions to ideal living conditions. Therefore, the variables that will be “happier than before” compared with their previous state and the state of other elderly people around the surrounding areas “happier than the surrounding people” are used as interpreted variables to verify reverse intergenerational support on the role of subjective well-being for the elderly. Second, change the measurement method. Replace the orderly Logit model with OLS, Ordered Probit model, Generalized Ordered Logit model, and Propensity Score Matching (PSM) model. There was no difference in the results of using both OLS and ordered Logit in a large sample case, and the estimation results of the OLS model were more intuitive and economical. The Probit model is used because its setting of the distribution of e is different from Logit. The results of the above robustness analysis are shown in columns (2) to (5) of Table 3. The reason for using Generalized Ordered Logit is that in the traditional Orderly Logit model, the estimation value of the parameter will not be changed with the level of well-being of the elderly, that is, $\beta_{i}$ is fixed between different levels of happiness. However, the General Order Logit model relaxes the parallel line assumption conditions. Assuming that the independent variable coefficient between the five levels of happiness may change, it takes into account the influence of the interpreted variables on its heterogeneity under different thresholds. There is a broad sense that has a broad sense. Seeing Logit return results are shown in Table 4. The reason for using PSM is that the presence or absence of reverse intergenerational support behaviors in older adults is not generated by random grouping and is prone to systematic bias. PSM can eliminate the interference factors between the groups, and solve the systematic deviation. The results are shown in Table 5.

**Table 4:** Regression results of the General Ordered Logit model.

	Reverse economic support	Reverse time support	Reverse spiritual support
Happiness level1→2	−0.554^**^	0.619^**^	0.441^**^
	(−2.036)	(2.104)	(2.086)
Happiness level2→3	−0.956^***^	0.403^**^	0.560^***^
	(−3.112)	(2.377)	(2.987)
Happiness level3→4	−0.617^***^	0.177^**^	0.374^***^
	(−4.525)	(2.450)	(4.178)
Happiness level4→5	−0.107^**^	0.166^**^	0.248^***^
	(−2.053)	(2.552)	(2.808)
Controlled variable	Control	Contro**l**	Control
N	3,170	3,170	3,170
*R*^2^	0.1757	0.1722	0.1751

^***^p < 0.01; ^**^p < 0.05; ^*^p < 0.1, as in the following tables.

**Table 5:** Average treatment effect on the treated (ATT) using PSM.

	Reverse economic support	Revers time support	Reverse spiritual support
Nearest neighbor matching (*K* = 4)	−0.179^**^	0.165^***^	0.233^***^
	(−0.071)	(0.027)	(0.061)
Kernel matching	−0.109^**^	0.184^**^	0.305^***^
	(−0.049)	(0.042)	(0.074)
*N*	3,170	3,170	3,170

^***^p < 0.01; ^**^p < 0.05; ^*^p < 0.1, as in the following tables.

Table 4, from columns (2) to (5), is to replace the explained variables with “happier than before” and “happier than others,” and the measurement method is replaced with the OLS and Probit model. Table 4 uses the Generalized Ordered Logit model. Among the elderly with different levels of happiness, the three types of reverse intergenerational support still have a significant effect on their happiness of the elderly. Table 5 adopts the PSM model. Based on the common support assumption that meets the tendency score matching, the processing effect is calculated with the most adjacent matching and nuclear matching. The results of matching the parallel inspection of the front and rear are as follows:$R^{2}$ are 0.015 and 0.001 before and after matching, respectively; The standardization deviation of the coordinating variable is 7.6 and 1.8 before and after the matching, respectively. Therefore, as far as the distribution of the collaborative variables between the two sets of samples, the tendency score estimation and the sample matching are successful. Also, the PSM model can address the endogeneity problem due to the selection bias of the sample.

The results of various stability tests are consistent with the direction of the basic regression results in the direction of the coefficient, which proves the stability of the regression test and verifies Hypothesis 1.

The Logit and Probit models report pseudo *R*^2^, as in the following tables.

## 6. Moderating effects

To verify whether the process of reverse intergenerational support for the elderly changes their happiness in the face of role conflict, this section will merge the groups with role enhancement in reverse economic support and groups with role conflict in reverse time support and reverse spiritual support, respectively, based on the previous analysis, as shown in Table 6. Through the analysis of these groups with role enhancement or role conflict, the coefficients of reverse intergenerational support were observed to change in direction and significance compared to the overall regression results, and the moderating effects of role conflict and role enhancement in them were found, as a way to test Hypotheses 2 and 3.

**Table 6:** Group division where role enhancement and role conflict exist.

Role enhancement/role conflict	Different characteristics of the elderly groups
Role-enhancing groups in reverse economic support	(1) Urban elderly living with urban children. (2) With only one child. (3) Without chronic diseases. (4) With positive intergenerational support
Role conflict groups in reverse time support	(1) Rural elderly living with urban children. (2) Aged 60–69. (3) With multiple children. (4) Chronic illnesses. (5) Delayed retirement. (6) No positive intergenerational support
Role conflict groups in reverse spiritual support	(1) Having multiple children. (2) No positive intergenerational support

The results of analyzing the role of reverse intergenerational support on the happiness of the above three different groups composed of elderly people with different characteristics are shown in Table 7. in column (1) where there is role enhancement, it can be found that the coefficient of the effect of their reverse economic support on happiness, although still negative, is no longer significant, which verifies Hypothesis 2, that when elderly people provide reverse economic support in the process of not having role conflict and achieving role enhancement, then the significant negative effect on happiness will also be dissolved. In the group with role conflict in column (2) reverse time support, the effect of reverse time support on the happiness of the elderly in this group is no longer significant, and in the group with role conflict in column (3) reverse spiritual support, the effect of reverse spiritual support on the happiness of the elderly in this group is also no longer significant, which verifies Hypothesis 3, that is when the elderly in the process of providing reverse time support and reverse spiritual support, if there is role conflict, the significant positive effect on happiness would be inhibited.

**Table 7:** Effect of reverse intergenerational support on the happiness of the elderly under role conflict/role enhancement.

	(1) Role-enhancing groups in reverse economic support	(2) Role conflict groups in reverse time support	(3) Role conflict groups in reverse spiritual support
Reverse economic support	−0.313	−0.264^**^	−0.403^**^
	(−0.936)	(−2.436)	(−2.240)
Reverse time support	0.242^**^	0.141	0.181
	(2.672)	(1.508)	(0.627)
Reverse spiritual support	0.294^***^	0.266^**^	0.254
	(3.300)	(2.181)	(1.387)
Controlled variable	Control	Control	Control
*N*	1,914	2,133	457
*R* ^2^	0.1682	0.1650	0.1338

^***^p < 0.01; ^**^p < 0.05; ^*^p < 0.1, as in the following tables.

## 7. Discussion and conclusion

Reverse intergenerational support is a prevalent social phenomenon in contemporary China in the wake of changing intergenerational relationships, and happiness is a valuable guide for government policies. Therefore, the current study collected 3,170 valid data and analyzed the effect of reverse intergenerational support on the happiness of Chinese older adults and the moderating effect of role conflict in it through the Logit model. It was found that reverse economic support reduced older adults’ well-being, but role reinforcement could dissolve the significant effect of reverse economic support on the happiness of the elderly. Reverse time support and reverse spiritual support increased the happiness of the elderly, but the presence of role conflict suppressed it.

This paper contributed to two strands of the literature. On the one hand, it is well known that China is a society that emphasizes traditional filial piety and has always emphasized feedback from adult children to their aging parents. This paper shifts the research perspective from traditional positive intergenerational support, i.e., intergenerational support from children to their parents, to emerging reverse intergenerational support. This enriches the factors influencing the happiness of the elderly in contemporary China. On the other hand, the introduction of role conflict theory to reverse intergenerational support is an innovative exploration, and role conflict provides a powerful explanation for the different feelings of happiness obtained by the elderly when providing reverse intergenerational support.

The established literature has started to try to introduce heterogeneity analysis to obtain a more in-depth observation in the examination of reverse intergenerational support and older adults’ well-being, such as financially well-off versus financially less well-off (Zheng et al., 2018), highly educated versus less educated (Mahne and Huxhold, 2015), working versus already retired (Arpino and Bellani, 2022), urban versus rural (Jin and Liu, 2017), and so on. The studies that have been conducted provide a good basis for subsequent research and based on this, there are two possible directions for future research on reverse intergenerational support and the happiness of the elderly: one is to continue to explore possible indicators to segment older adult groups and examine the relationship between respective reverse intergenerational support and their happiness; the other is to apply a theoretical framework to provide a more general explanation for the unity of heterogeneity in reverse intergenerational support. This paper makes an attempt in the second direction by introducing role conflict theory into the analysis. On the one hand, the results obtained corroborate with the findings of previous scholars in many localized areas, such as the effects of grandchild care and work participation on the well-being of female older adults (Arpino and Bellani, 2022). On the other hand, stepping out of the limitations oriented toward a local domain enables a more global perspective to examine the heterogeneity of well-being among older adults in reverse intergenerational support.

Based on the findings of this paper, to improve the happiness of Chinese elderly people, on one hand, we need to pay attention to the prevalent phenomenon of reverse intergenerational support. Combined with the results of the basic regression, in terms of reverse economic support, we should eliminate the “intergenerational support exploitation” or “obligation theory” of some young offspring, to reduce the pressure of reverse economic support from the parents. In terms of reverse time support, we should eliminate regional barriers and realize the connection between outflow and inflow places in terms of services. In terms of reverse time support, we should eliminate regional barriers and realize the docking of services between outflow and inflow places, to encourage co-living or living nearby and create the support conditions for reverse time support; in terms of reverse spiritual support, we should promote filial piety culture to realize spiritual support from children to parents and create two-way intergenerational relationships; on the other hand, it is also necessary to pay attention to the role conflict that may appear behind the reverse intergenerational support, and in combination with the role conflict regulation effect, efforts should be made to resolve the role conflict that may appear. In terms of expectation conflict, we should focus on family education and family style construction, and inherit and promote traditional filial culture in various forms, so that the children can fulfill their support obligations more effectively and realize the two-way exchange under the intergenerational relationship. In terms of capacity conflicts, deepening the multi-pillar old-age security system, especially for the rural elderly, allows the elderly to have a stronger ability to reverse economic support with adequate economic security, and allows the elderly to have a stronger ability to reverse time support with healthier physical conditions. In terms of time conflict, a flexible delayed retirement system should be established. The state can provide institutional incentives for delayed retirement and impose certain constraints on early retirement, but allow the elderly to make their own retirement decisions according to their family’s actual situation. At the same time, we should develop a childcare system so that the elderly can participate in useful social activities according to their wishes. In terms of behavioral conflicts, we should help the rural elderly who move to other places to integrate into urban life faster and better and strengthen the friendly mechanism for the elderly who move with them in the construction of age-friendly cities and friendly communities. By resolving the above role conflicts, we can effectively improve the happiness of contemporary Chinese elderly people.

Some limitations of this study should be noted. First, the conclusion of this study comes from the unique cultural background of China. Although it can provide a reference for other countries or regions, it does not mean that the conclusion is still valid for other countries and regions, which requires further analysis and research based on local data. Second, it is difficult to directly measure the role conflict that exists in reverse intergenerational support for older adults, so this paper uses grouping to examine role conflict and uses it as a moderating variable for mechanism analysis. In the future, when a precise evaluation of role conflict is achieved, the effect of role conflict on the happiness of the elderly can be further examined.

## Data availability statement

The raw data supporting the conclusions of this article will be made available by the authors, without undue reservation.

## Ethics statement

Ethical review and approval was not required for the study on human participants in accordance with the local legislation and institutional requirements. Written informed consent from the participants was not required to participate in this study in accordance with the national legislation and the institutional requirements.

## Author contributions

All authors listed have made a substantial, direct, and intellectual contribution to the work and approved it for publication.

## Funding

This study was supported by the reserach project “Jiangsu Overseas Visiting Scholar Program for Univetsity Prominent Young & Middle-aged Teachers and Presidents”.

## Conflict of interest

The authors declare that the research was conducted in the absence of any commercial or financial relationships that could be construed as a potential conflict of interest.

## Publisher’s note

All claims expressed in this article are solely those of the authors and do not necessarily represent those of their affiliated organizations, or those of the publisher, the editors and the reviewers. Any product that may be evaluated in this article, or claim that may be made by its manufacturer, is not guaranteed or endorsed by the publisher.
